# Distinct patterns of serum and urine macrophage migration inhibitory factor kinetics predict death in sepsis: a prospective, observational clinical study

**DOI:** 10.1038/s41598-023-27506-6

**Published:** 2023-01-11

**Authors:** Janos Toldi, Leonardo Kelava, Sandor Marton, Diana Muhl, Peter Kustan, Zsolt Feher, Klaudia Maar, Janos Garai, Eszter Pakai, Andras Garami

**Affiliations:** 1 Department of Thermophysiology, Institute for Translational Medicine, Medical School, University of Pecs, Pecs, Hungary; 2 Department of Anesthesiology and Intensive Care, Medical School, University of Pecs, Pecs, Hungary; 3 Department of Laboratory Medicine, Medical School, University of Pecs, Pecs, Hungary; 4 Department of Biochemistry and Medical Chemistry, Medical School and Research Group of Regenerative Science, Sport and Medicine, Janos Szentagothai Research Centre, University of Pecs, Pecs, Hungary; 5 Department of Pathophysiology, Institute for Translational Medicine, Medical School, University of Pecs, Pecs, Hungary

**Keywords:** Predictive markers, Prognostic markers, Sepsis

## Abstract

Macrophage migration inhibitory factor (MIF) has been considered as a biomarker in sepsis, however the predictive value of the pattern of its kinetics in the serum and in the urine has remained unclarified. It is also unclear whether the kinetics of MIF are different between males and females. We conducted a single-center prospective, observational study with repeated measurements of MIF in serum and urine on days 0, 2, and 4 from admission to the intensive care unit (ICU) in 50 adult septic patients. We found that in patients who died within 90 days, there was an increase in serum MIF level from day 0 to 4, whereas in the survivors there was rather a decrease (*p* = 0.018). The kinetics were sex-dependent as the same difference in the pattern was present in males (*p* = 0.014), but not in females (*p* = 0.418). We also found that urine MIF was markedly lower in patients who died than in survivors of sepsis (*p* < 0.050). Urine MIF levels did not show temporal changes: there was no meaningful difference between day 0 and 4. These results suggest that kinetics of serum MIF during the initial days from ICU admission can predict death, especially in male patients. Additionally, lower urine MIF levels can also indicate death without showing meaningful temporal kinetics.

## Introduction

Sepsis is a life-threating disease that develops when the host immune response to an infection becomes dysregulated, thereby it damages its own tissues and organs^1^. The global burden of sepsis constitutes a challenge for the patients and healthcare personnel, which is also indicated by the high incidence of hospital-treated sepsis cases across all regions (189/100,000 person years) reported in 2020^2^. Moreover, the estimated death rate in septic patients was as high as 26.7%, which was further increased to 41.9% when the patients were treated at the intensive care unit (ICU)^2^. In the same year, another study concluded that the estimated burden of sepsis worldwide is twice as much as what was thought previously^3^. Further increasing its burdens, sepsis was also associated with greater rehospitalization rates and higher healthcare costs compared to matched hospitalized controls according to a recent study^4^. The early diagnosis and assessment of severity could reduce the burdens of sepsis, which can be achieved through the discovery of reliable biomarkers.

In our recent meta-analysis, we showed that the blood level of macrophage migration inhibitory factor (MIF), a pro-inflammatory and immunoregulatory cytokine^5,6^, can be a valuable diagnostic and prognostic biomarker in sepsis^7^. We found that blood MIF levels were higher in septic patients who had more advanced severity and did not survive the disease. However, in most of the analyzed studies, the blood MIF level was measured only once on a single day in the patients, which did not allow us to assess the temporal kinetics of blood MIF level during the progression of sepsis and its association with the outcome of the disease. We identified only three studies, in which blood MIF levels were measured and reported on at least two days in sepsis survivors and nonsurvivors^8–10^, which were not suitable for proper meta-analysis. Importantly, their results were controversial. In one of those papers, there was no meaningful difference in blood MIF levels between days 0, 2, and 5 in sepsis survivors (*p* = 0.196) and nonsurvivors (*p* = 0.105)^8^, whereas in another study, high incremental increases in blood MIF levels between days 1 and 2 were associated with higher mortality in severely septic patients^9^. In the third study, serum MIF level seemed to increase on days 0 and 1 of the study, then it decreased by day 10 in both survivors and nonsurvivors, however the statistical analysis of the temporal changes was not reported^10^.

The kinetics of a biomarker incorporates the time-dependent changes in the synthesis, metabolism, and elimination of the substance, which can show variations during the progression of a disease. In accordance, the importance of biomarker kinetics has been recognized in sepsis, although its low investigation rate compared to single time-point measurements was also noted^11,12^. For instance, the kinetics of plasma procalcitonin was superior to a single measurement for the prediction of death in septic patients^13^. Similarly, the time-dependent change in blood heparin‑binding protein level was more accurate than its initial value for prediction of the fatal outcome in sepsis^14^.

In addition to the blood levels of MIF, urine MIF may also serve as a useful biomarker in inflammatory diseases^6^, but to our knowledge its potential value as a predictor of the outcome in sepsis has not been investigated. Some studies showed a correlation between urine MIF levels and kidney injury in infectious acute pyelonephritis^15,16^, in glomerulonephritis^17^, and in renal transplant rejection^18^, which may suggest that urine MIF could be a useful predictive parameter of renal dysfunction, but data on urine MIF kinetics in septic patients could not be found in the literature.

In the present study, we aimed at determining the kinetics of blood and urine MIF levels in septic patients during the initial days from their admission to the ICU at the University of Pecs, Hungary.

## Methods

### Patients

Between January 2012 and May 2015, we enrolled 51 septic patients into this prospective, observational study from our ICU (Department of Anesthesiology and Intensive Therapy, University of Pecs, Pecs, Hungary). Our study protocol was approved by the Regional Research Ethical Committee of the University of Pecs (registration no.: 2406/2005; full date of first registration: 01/04/2005) and the study was performed in accordance with the ethical standards in the 2008 Declaration of Helsinki. Due to the pure observational nature of our study, further registration was not required according to the recommendations of the International Committee of Medical Journal Editors (ICMJE). Following the detailed explanation of the study procedure, written informed consent was obtained from all study participants. All methods were performed in accordance with the relevant guidelines and regulations.

### Inclusion and exclusion criteria

Sepsis was defined according to the criteria of the 2001 International Sepsis Definitions Conference^19^. Septic patients with elevated serum procalcitonin level at admission to the ICU were enrolled in the study. Patients were excluded if they were under 18 years or above 85 years of age or if they refused to participate in the study. Except for the measurements of MIF levels, the diagnostic and treatment procedures were conducted according to the sepsis guidelines in the patients.

### Data collection

Demographic data on age and sex were collected from all enrolled patients. Mortality was followed up for 90 days from ICU admission. Laboratory parameters including serum concentrations of C-reactive protein, procalcitonin, lactate, urea, and creatinine, as well as blood cell counts were measured on days 0, 2, and 4 from ICU admission. On the same days, the urine concentrations of creatinine and total protein, as well as the estimated glomerular filtration rate were also determined. The Sequential Organ Failure Assessment (SOFA) score^20^, the Simplified Acute Physiology Score (SAPS) II^21^, and the Acute Physiology and Chronic Health Evaluation (APACHE) II score^22^ was calculated on admission to the ICU. Renal dysfunction was defined as more than 50% increase in serum creatinine levels above the baseline according to the RIFLE (acronym indicating Risk of renal dysfunction; Injury to the kidney; Failure of kidney function, Loss of kidney function, and End-stage kidney disease) criteria^23^.

### Measurement of MIF concentration

Urine and venous blood samples were collected for the measurements of MIF levels on days 0, 2, and 4 from ICU admission. Blood was collected in Vacutainer serum tubes with silicon coating as clot accelerator (Becton, Dickinson and Company, Franklin Lakes, NJ, USA), and it was kept in the tubes at room temperature to clot for at least 60 min. Serum was collected after centrifugation at 1300 g for 10 min at room temperature, then it was aliquoted and stored at −70°C until the analysis. The levels of MIF were measured in urine and serum by using standard enzyme-linked immunosorbent assay (ELISA) kits (catalog number: DY289; R&D Systems, Minneapolis, MN, USA) according to the manufacturer's recommendations as in a previous study^24^. All measurements were performed in duplicates. The plates were read at 450 nm by using an iEMS MF microphotometer (Thermo Labsystem, Beverly, MA, USA).

The rational for using serum samples was that serum MIF measurements were successfully used to investigate the biomarker role of MIF in septic patients by different authors before patient recruitment started into our study^9,25^. Although the use of serum (instead of plasma) MIF was later criticized^26^, another study showed no significant difference between plasma and serum cytokine levels, including MIF^27^, while more recently the use of serum rather than plasma samples for MIF detection was recommended in clinical studies to prevent interference from anticoagulants and maintain the consistency of research^28^. Nevertheless, since we used the same sample type in all patients in the present study, the quantitative comparisons of the patient groups should be appropriate based on prior recommendations^29^.

When studying renal dysfunction, the levels of urine MIF were also calculated as ratios relative to the urine creatinine level based on earlier studies^15,16^.

### Statistical analysis

The statistical analysis of the collected data was performed with the R software (version 3.6.1; R Development Core Team, Vienna, Austria). The Mann-Whitney test was used to detect significant differences in urine and serum MIF levels between survivors and nonsurvivors. In subgroup analysis, repeated measures ANOVA was performed with either serum MIF or urine MIF as a dependent variable, while time and either sex or age were the independent variables. To analyze whether temporal changes of MIF levels during sepsis can have significant predictive value, the change in serum MIF from day 0 to day 4 was calculated, and then the change was compared between survivors and nonsurvivors with the Mann-Whitney test. Frequency tables for deaths were generated in groups with different patterns of MIF kinetics, and then the number of deaths were compared with the Fisher test between the groups. The data are reported in the mean ± standard error (SE) format, unless specified otherwise.

## Results

### Patient characteristics

**Figure 1:**
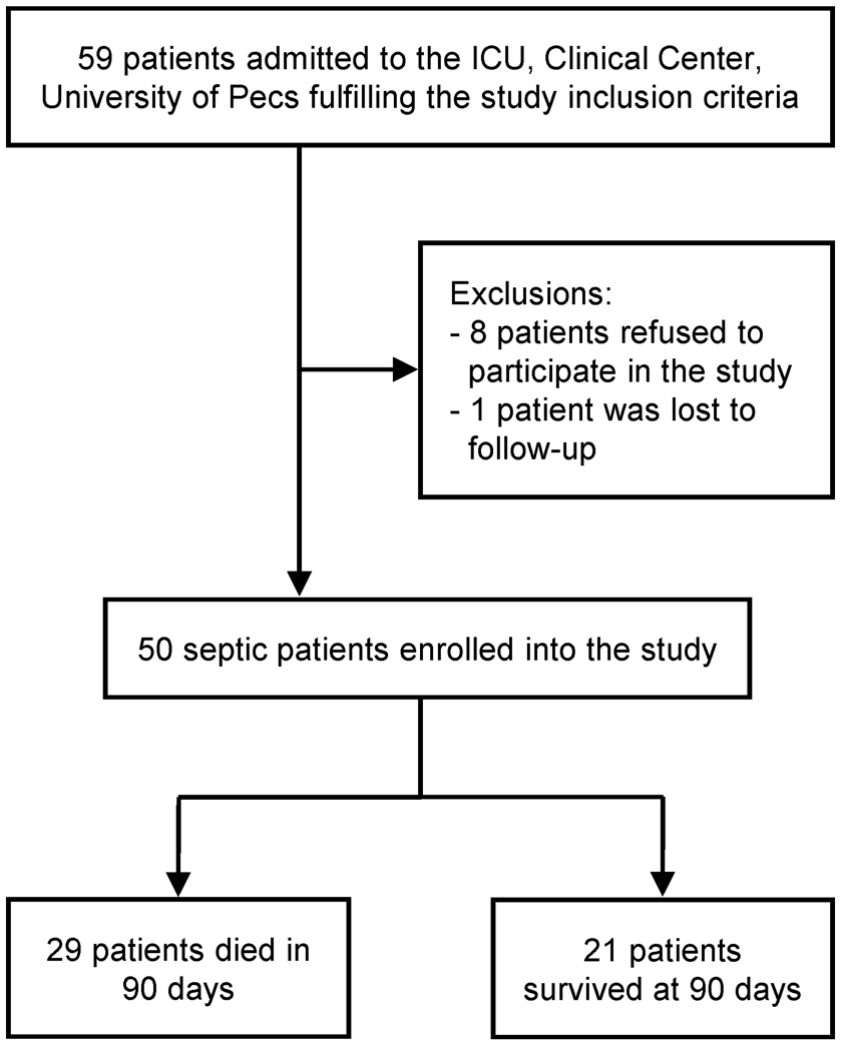
Flow diagram of the study.

During the study period, 59 patients were eligible for the study according to the inclusion criteria, but only 51 patients could be enrolled, because 8 of them refused to participate after they received detailed information about the study. One patient had to be excluded, because the outcome could not be recorded at the end of the 90-day follow up. We included data from 50 patients in the final analysis. The flow diagram of the study is shown in Fig. 1. In the included patient population, sepsis was diagnosed post-surgically in 33 cases (25 after acute and 8 after elective surgical interventions), while in the remaining cases without a preceding surgery, pneumonia (n = 10), pancreatitis (n = 2), urosepsis (n = 1), erysipelas (n = 1), and unidentified initial infections (n = 3) were associated with sepsis.

The baseline characteristics of the 50 patients analyzed in the study are summarized in Table 1. The statistical comparison of all parameters between survivor (n = 21) and nonsurvivor (n = 29) groups is also included in the table. The 90-day mortality rate was 58% in this study population, which is comparable with recent data reported in the literature^30^. The sex and age distribution of the patients were similar in the two groups, so was the number of cases with renal dysfunction as assessed by the RIFLE criteria^23^. Importantly, on the day of admission to the ICU, we did not detect a significant difference in any parameters between the two groups, although, the SAPS II and SOFA scores tended to be higher in nonsurvivors than in survivors (*p* = 0.15 and 0.16, respectively), as it could be expected.

**Table 1:** Basic demographic data, laboratory parameters, and clinical scores of the survivor and nonsurvivor septic patients on the admission day to the intensive care unit.

Parameters (unit)	Survivors (n = 21)	Nonsurvivors (n = 29)	All (n = 50)	*p* value
**Demographic characteristics**				
Age (years)	67 ± 3	66 ± 3	66 ± 2	0.78
65 years old or older, n (%)	12 (57)	17 (59)	29 (58)	1.00
Female, n (%)	12 (57)	11 (38)	23 (46)	0.57
**Blood test results**				
Red blood cell count (10^12^/l)	3.7 ± 0.1	3.5 ± 0.1	3.5 ± 0.1	0.17
White blood cell count (10^9^/l)	13.6 ± 0.2	15.1 ± 2.1	14.5 ± 1.5	0.63
Neutrophil percentage (%)	12 ± 2	14 ± 2	13 ± 1	0.68
C-reactive protein (mg/l)	232.4 ± 28.1	253.5 ± 21.8	244.7 ± 17.2	0.56
Procalcitonin (ng/ml)	23.62 ± 10.99	40.22 ± 10.19	33.86 ± 7.60	0.27
Lactate (mmol/l)	4.4 ± 1.3	4.0 ± 1.3	4.2 ± 0.9	0.81
Creatinine (µmol/l)	180.2 ± 31.0	173.8 ± 23.1	176.5 ± 18.5	0.87
Urea (mmol/l)	13.7 ± 1.8	15.6 ± 1.7	14.8 ± 1.2	0.47
Estimated glomerular filtration rate (ml/min/1.73 m^2^)	37.6 ± 5.1	39.6 ± 4.4	38.8 ± 3.3	0.78
Urine test results*				
Total protein (mg/l)	1193 ± 642	713 ± 213	856 ± 240	0.49
Creatinine (mmol/l)	4.5 ± 1.0	5.2 ± 0.8	5.0 ± 0.6	0.61
**Clinical status evaluation**				
APACHE II (score)	17 ± 2	19 ± 2	18 ± 1	0.39
SAPS II (score)	40 ± 4	49 ± 4	46 ± 3	0.15
SOFA (score)	8 ± 1	10 ± 1	10 ± 1	0.16
Renal dysfunction, n (%)	13 (62)	16 (55)	29 (58)	0.77

*urine samples for the present study could not be obtained on the day of admission from 6 patients (1 survivor and 5 nonsurvivors). Data are expressed as mean ± standard error, except for the sex, elderly, and renal dysfunction ratio, where number (and percentage) of patients is shown. *APACHE*, acute physiology and chronic health evaluation score; *SAPS*, simplified acute physiology score; *SOFA*, sequential (sepsis-related) organ failure assessment score.

### Serum and urine MIF levels in sepsis on the initial days from ICU admission

**Figure 2:**
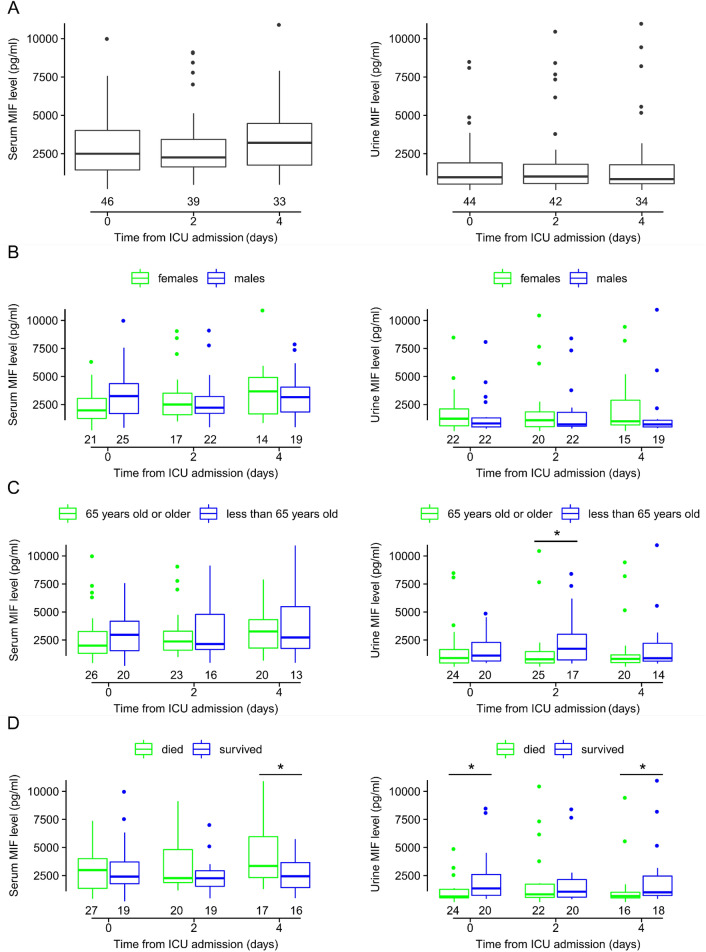
The serum and urine levels of macrophage migration inhibitory factor (MIF) in septic patients on days 0, 2, and 4 from admission to the intensive care unit (ICU). The MIF levels are shown in (**A**) all patients and in patient subgroups of (**B**) females and males, (**C**) at least 65 years old and younger than 65 years, and (**D**) deceased and survived. Here, and in Fig. 6A, the horizontal line within each box represents the median, the bottom and the top of the box marks the lower and the upper quartile, respectively, which limit the interquartile range (IQR). The vertical line below and above the box shows the minimum and maximum levels, respectively. Outliers are shown with dots. The numbers below the boxes indicate the number of patients in each group. Note that on day 0, serum MIF level could not be determined in 4 patients and urine MIF level in 6 patients due to technical issues. *p < 0.05.

Figure 2A shows the median levels of serum and urine MIF in all septic patients on days 0, 2, and 4 from admission to our ICU. On all days, the MIF levels were higher in the serum than in the urine with medians (and interquartile range, IQR) of 2500 (1441–4015), 2255 (1638–3432), and 3209 (1761–4470) pg/ml in serum versus 965 (520–1905), 1013 (561–1813), and 845 (541–1783) pg/ml in urine, on day 0, 2, and 4, respectively. As in earlier studies^15,16^, we also normalized urine MIF levels to urine creatinine, which did meaningfully impact the observed kinetics: the medians and (IQRs) of the urine MIF/creatinine ratios on day 0, 2, and 4 were 0.30 (0.15–1.34), 0.54 (0.19–1.28), and 0.29 (0.16–0.80) pg/µmol, respectively. The medians were not statistically different between the days either in the serum or in the urine samples, even though there was a 28% increase in serum MIF from day 0 to day 4. The minimum and maximum serum MIF levels were also the highest on day 4 (478 and 7902 pg/ml, respectively).

The influence of sex and age on the progression of sepsis was proposed in previous studies^31–33^. Therefore, next we studied whether the serum and urine MIF kinetics observed in all patients remain similar when the patients are divided into subgroups based on sex (Fig. 2B), age (Fig. 2C), and survival (Fig. 2D). We did not find statistical difference between males and females in serum and urine MIF levels on any of the days. In females, the median serum MIF levels were 1979, 2495, and 3676 pg/ml on day 0, 2, and 4, respectively, while in males the medians were 3252, 2217, and 3163 pg/ml on the respective days (Fig. 2B). Urine MIF levels did not change meaningfully over time in either of the sexes. On all days the levels seemed somewhat higher in females than in males, but the intersex difference did not reach the level of significance. The urine MIF/creatinine ratio did also not change meaningfully over time in either sex, and it was not significantly different between females and males on any of the days.

When patients were divided into younger (less than 65 years old) and older groups (65 years old and above), serum MIF levels in the older patient group were 2000, 2368, and 3263 pg/ml on day 0, 2, and 4, respectively. In the younger patient group, the medians on the respective days were 2969, 2142, and 2732 pg/ml. There was no significant difference between the age groups on any of the days. The urine MIF levels did not differ meaningfully in the elderly between the days, while in the younger patients there was an increase from day 0 to day 2 reaching a median of 1722 pg/ml (vs. 782 pg/ml in the elderly; *p* = 0.028), then it decreased to similar median (871 pg/ml) as in the elderly (819 pg/ml) on day 4 (Fig. 2C). The urine MIF/creatinine ratio was not significantly different between younger and older patients on any of the days, and it did not change markedly over time in either age group. Since the ratio was not significantly different (*p* = 0.385) between younger and older patients on day 2 with respective medians (and IQRs) of 0.56 (0.35–1.22) pg/µmol and 0.32 (0.19–1.28) pg/µmol, these results indicate that the difference in urine MIF between the age groups on day 2 (Fig. 2C) was probably due to a difference in general kidney functions and not due to a difference specifically in MIF excretion.

The median serum MIF levels did not differ statistically between survivors and nonsurvivors on days 0 and 2, however on day 4 serum MIF was significantly (*p* = 0.039) higher in patients who died than who survived with medians (and IQRs) of 3348 (2313–5961) and 2430 (1284–3691) pg/ml, respectively (Fig. 2D). These results suggested different kinetics of serum MIF from day 0 to day 4 between survivors and nonsurvivors of sepsis. With regards to urine MIF, the medians did not change meaningfully over time in either of the subgroups. However, urine MIF levels were lower in patients who died than who survived on all days, which difference was significant on day 0 (638 vs. 1355 pg/ml; *p* = 0.046) and on day 4 (672 vs. 1005 pg/ml; *p* = 0.032). Similar to urine MIF, the urine MIF/creatinine ratio was not significantly different between the days in either subgroup. More importantly, as in the case of urine MIF, the significant differences in the ratio were also detectable between nonsurvivors and survivors on day 0 (0.24 vs. 0.50 pg/µmol; *p* = 0.022) and on day 4 (0.24 vs. 0.80 pg/µmol; *p* = 0.003). These findings suggest that the observed differences in urine MIF levels between survivors and nonsurvivors were presumably caused by differences specific to renal MIF excretion and not by differences in general renal functions.

### The kinetics of serum MIF levels in survivors and nonsurvivors of sepsis after ICU admission

**Figure 3:**
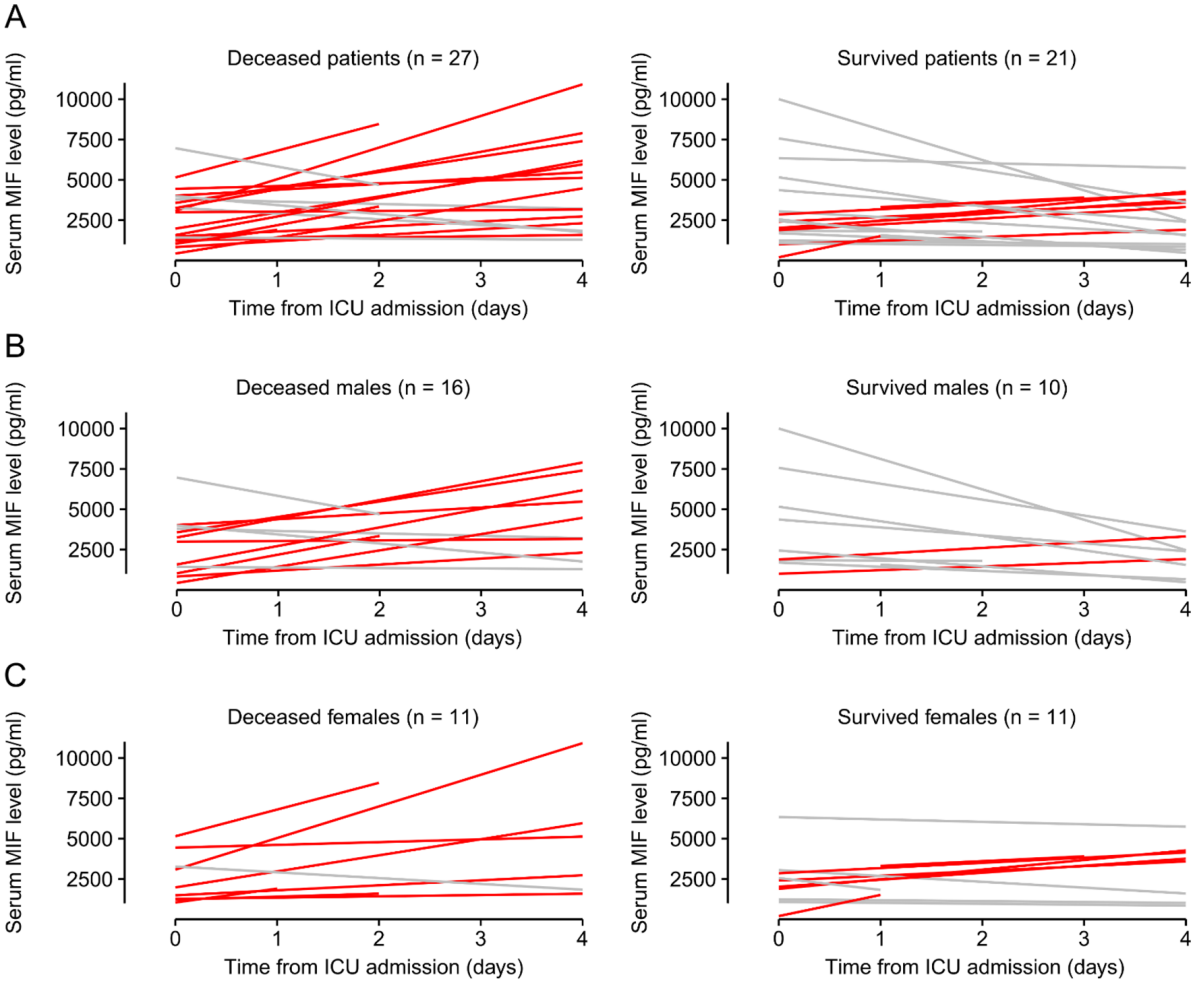
The individual pattern of serum macrophage migration inhibitory factor (MIF) kinetics in each patient who had at least 2 measurements between day 0 and 4 at the intensive care unit (ICU). Red line indicates an increase, while gray line shows no increase in serum MIF level in deceased and survived patients based on data obtained from (**A**) both sexes, (**B**) females, and (**C**) males. The number of patients (n) is indicated in the figure in each group.

Serum MIF levels were significantly higher in nonsurvivors than in survivors on day 4, but they did not differ on the day of ICU admission (Fig. 2D). Thus, we analyzed how the serum MIF levels changed from the first until the last measurement in each individual patient, and then compared the kinetics between those who survived and who deceased in sepsis (Fig. 3A). We included only those patients who had at least two serum MIF level values on different days during their stay at the ICU (n = 48), while 2 patients could not be included, because they died before a second blood sample could be collected from them. We found that serum MIF level increased in 15 of 27 deceased patients (~ 56%), while in the rest it did not change (n = 7) or decreased (n = 5). In contrast with the dominance of the increasing pattern in the deceased patients, in the survivors the most common (~ 62%) trend was a decrease in serum MIF level (n = 13), while it increased only in 8 out of the 21 patients.

In previous studies, an association between MIF and estrogen was indicated in inflammatory conditions, since estrogen inhibited endotoxin-induced MIF production in murine macrophages^34^, and it decreased MIF production in rat models of colitis^35^ and trauma-hemorrhage-induced lung injury^36^. Furthermore, MIF plasma levels were positively correlated with testosterone and negatively with estradiol in human patients^37^. Therefore, we also studied the changes in serum MIF levels in males and females separately even though the subgrouping lowered the number of patients in the analyzed groups (Fig. 3B,C). In males, similar kinetic patterns were present as in all patients: the most common (50%) trend was an increase in deceased patients, while a decrease was the dominant (80%) trend in the survivors (Fig. 3B). In contrast with males, in females the kinetic patterns did not differ meaningfully between survivors and nonsurvivors. In females, an increase in serum MIF was the most frequent (~ 73%) in deceased patients, as well as in the survivors (~ 55%) (Fig. 3C).

**Figure 4:**
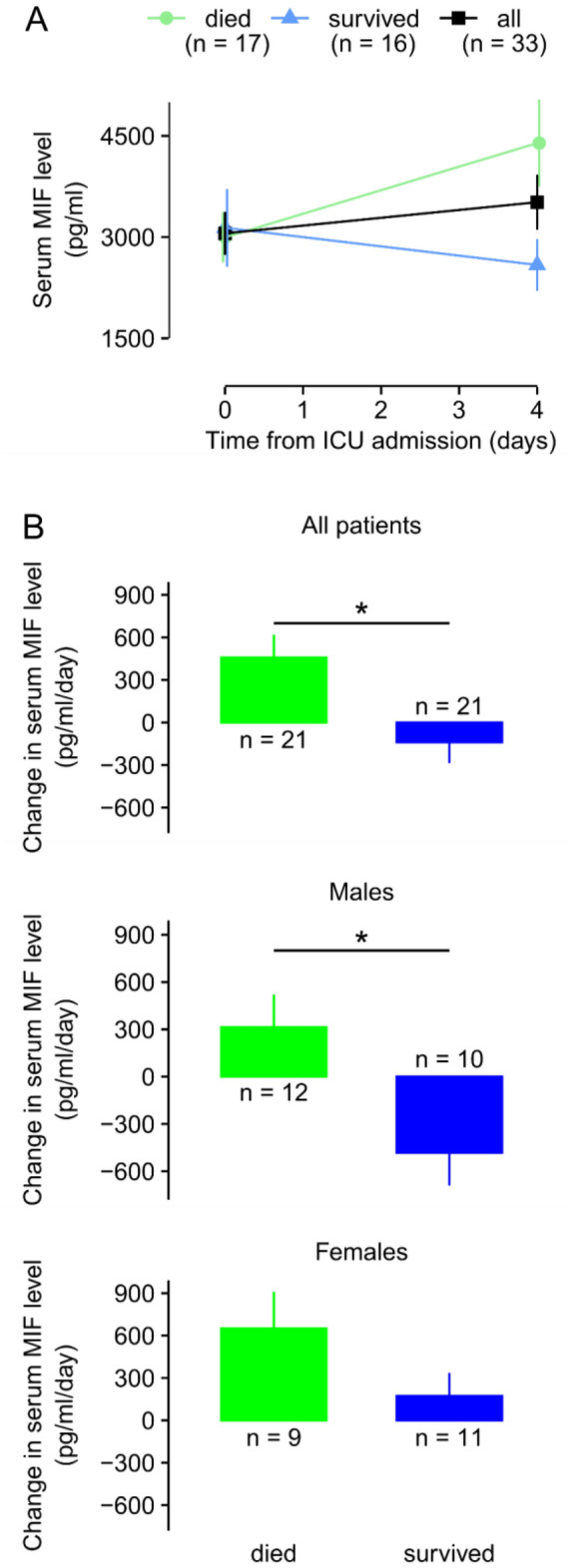
The kinetics of serum macrophage migration inhibitory factor (MIF) levels in septic patients at the intensive care unit (ICU). (**A**) The mean absolute serum levels of MIF in all, deceased, and survived septic patients on day 0 and 4 from admission to the ICU. (**B**) The mean daily changes of serum MIF levels in deceased and survived patients based on data obtained from both sexes (top), males (middle), and females (bottom). The number of patients (n) is indicated in the figure in each group. *p < 0.05.

For a more quantitative analysis of the serum MIF kinetics, in our next approach we also compared the mean changes of serum MIF levels between day 0 and 4 in all groups (Fig. 4). In accordance with our observations regarding the patterns of the kinetics, in deceased patients, the mean (± SE) serum MIF level increased from 2997 ± 373 pg/ml on day 0 to 4394 ± 646 pg/ml on day 4, whereas in the sepsis survivors serum MIF decreased from 3137 ± 576 to 2587 ± 384 pg/ml during the same time interval (Fig. 4A). The daily change in serum MIF level was significantly different between survivors and nonsurvivors, when we analyzed the data obtained from both sexes (*p* = 0.01) and from males (*p* = 0.01), whereas there was no marked difference between the deceased and survived groups in females (*p* = 0.230) (Fig. 4B). The previously observed patterns were also reflected by the mean daily changes in serum MIF, since an overall increase versus decrease was present in all and male nonsurvivors versus survivors, respectively, while in females there was on average an increase in both outcome groups.

### The kinetics of urine MIF levels in survivors and nonsurvivors of sepsis after ICU admission

**Figure 5:**
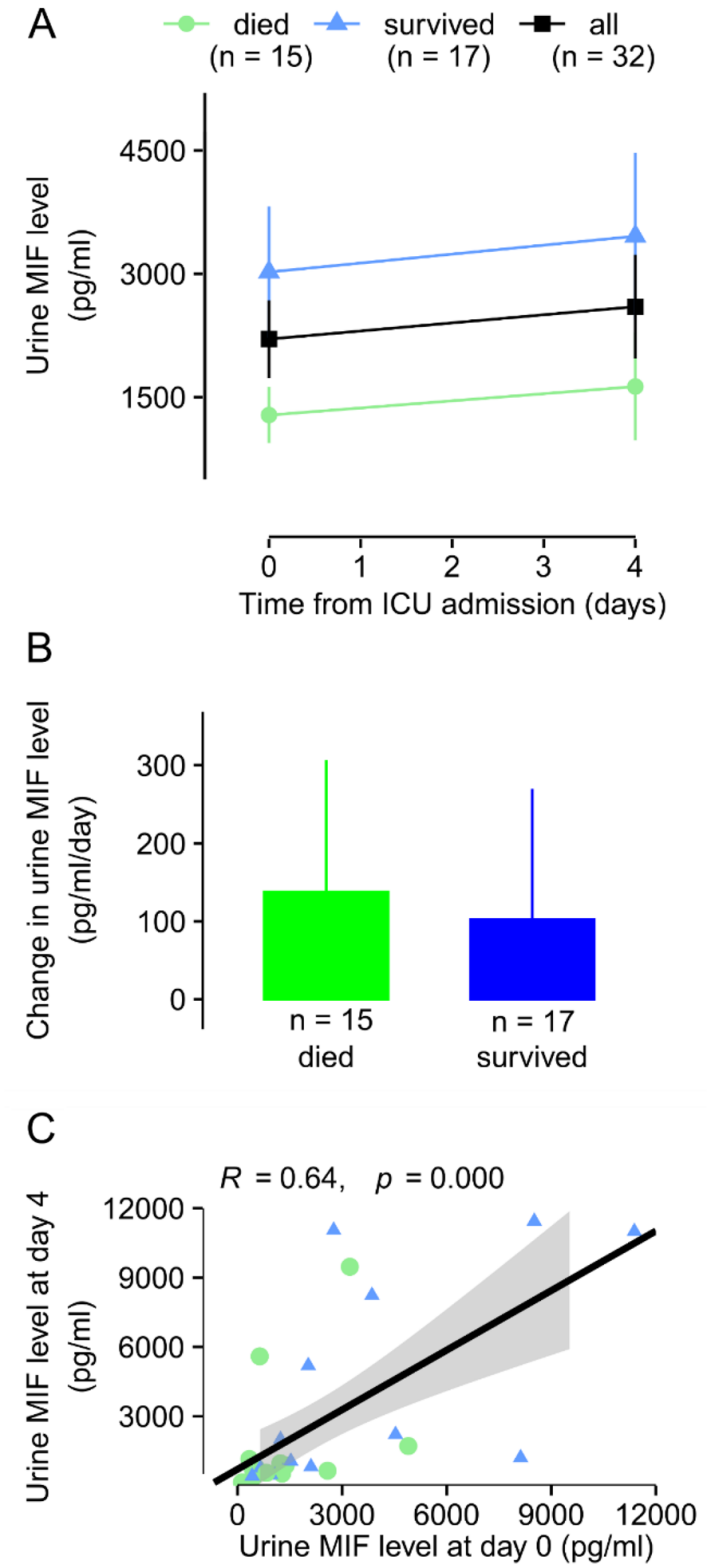
The kinetics of urine macrophage migration inhibitory factor (MIF) levels in septic patients at the intensive care unit (ICU). (**A**) The mean absolute urine levels of MIF in all, deceased, and survived septic patients on day 0 and 4 from admission to the ICU. (**B**) The mean daily changes of urine MIF levels in deceased and survived patients. (**C**) The correlation between urine MIF levels measured on day 0 and on day 4 from the admission to the ICU. The number of patients (n) is indicated in the figure in each group.

In addition to serum levels of MIF, we also studied how the levels of MIF change in the urine after the admission of the septic patients to the ICU. As mentioned before, the urine MIF levels were significantly lower in deceased patients than in survivors on days 0 and 4 (see Fig. 2D). When we looked at the kinetics within the groups, we found a small, not significant increase in both groups from day 0 to day 4: from 3021 ± 797 to 3457 ± 1016 pg/ml in survivors and from 1281 ± 340 to 1629 ± 654 pg/ml in nonsurvivors (Fig. 5A). Importantly, the daily change in the urine levels of MIF did not differ significantly between survivors and nonsurvivors (109 ± 192 vs. 87 ± 152 pg/ml; *p* = 0.940) (Fig. 5B). There was also no significant difference in the daily change of urine MIF levels between the outcome groups when we compared males and females separately (*p* = 0.136 and *p* = 0.228, respectively). By analyzing the data obtained from both sexes, we found a strong positive correlation between urine MIF levels measured on day 0 and on day 4 (Fig. 5C), suggesting that the level determined on day 0 can predict the value on day 4.

### The kinetics of urine MIF levels in septic patients with and without renal dysfunction after ICU admission

Some studies showed that urine MIF can be an indicator of renal dysfunction associated with different diseases^15–18^, but whether it has a similar indicator role in sepsis has remained unclear. Therefore, in our next approach we compared urine MIF levels in septic patients who developed renal dysfunction and in those who did not according to the RIFLE criteria^23^.

**Figure 6:**
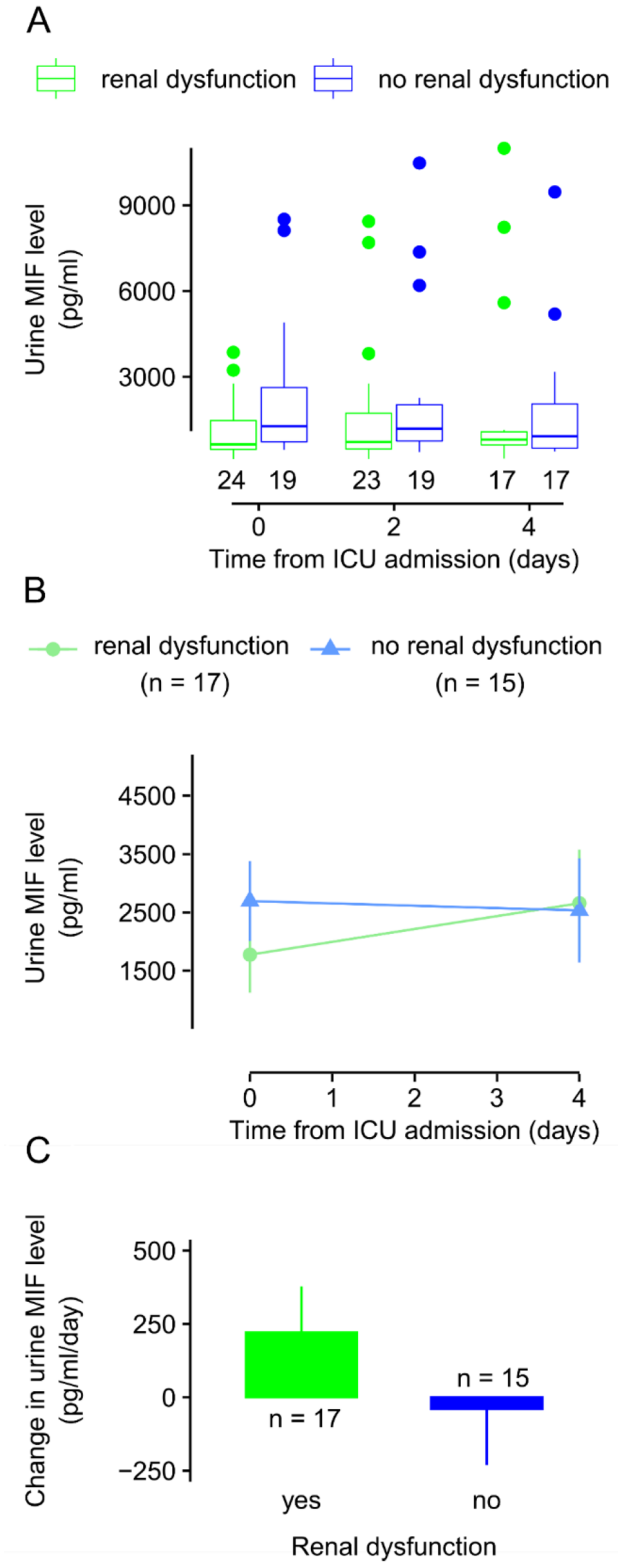
The kinetics of urine macrophage migration inhibitory factor (MIF) levels in septic patients with and without renal dysfunction at the intensive care unit (ICU). (**A**) Box plot of urine levels of MIF in septic patients with and without renal dysfunction on day 0, 2, and 4 from admission to the ICU (for explanation of symbols, see Fig. 2). (**B**) The mean absolute urine levels of MIF in septic patients with and without renal dysfunction on day 0 and 4 from admission to the ICU. (**C**) The mean daily changes of urine MIF levels in septic patients with and without renal dysfunction. The number of patients is indicated in the figure in each group.

The median urine MIF levels seemed higher in patients with healthy kidney functions than in those who had renal dysfunction on days 0, 2, and 4, which difference was the biggest on day 0 with medians (and IQRs) of 1268 (725–2626) pg/ml and 638 (461–1467) pg/ml, respectively (Fig. 6A). Importantly, however, the difference between the groups did not reach the level of significance on any of the days. Normalization of urine MIF levels to urine creatinine did not meaningfully impact the observed kinetics: the urine MIF/creatinine ratio seemed higher in patients without renal dysfunction on days 0 and 2 with the biggest difference in the medians (and IQRs) between patients with and without renal dysfunction on day 2: 0.29 (0.16–0.87) pg/µmol and 0.65 (0.28–1.88) pg/µmol. However, the difference was not statistically significant between the groups on any of the days.

Between day 0 and 4 from ICU admission, the urine MIF level changed on average from 2694 ± 686 to 2534 ± 893 pg/ml in patients without renal dysfunction, while from 1774 ± 653 to 2658 ± 918 pg/ml in patients with renal dysfunction (Fig. 6B). There was no significant difference between the groups. The mean daily changes in urine MIF levels were 220 ± 157 pg/ml and − 40 ± 191 pg/ml with and without renal dysfunction, respectively (Fig. 6C), which were not statistically different between the groups even if the urine MIF/creatinine ratios were used for comparison of the groups (0.01 ± 0.04 vs. − 0.01 ± 0.13 pg/µmol/day, respectively).

## Discussion

Here, we present the kinetics of serum and urine MIF levels in septic patients on the initial days from ICU admission. We show that the patterns of serum MIF kinetics are different between patients who survived and who died in sepsis. We report, to the best of our knowledge for the first time, that serum MIF level increased after ICU admission in those patients who died in sepsis, whereas it decreased in the survivors of the disease. With subgroup analysis, we detected intersex difference in the kinetics of serum MIF in sepsis, since the decreasing trend in the survivors was present in males, but not in females. Moreover, we show that urine MIF level can be a valuable prognostic marker of mortality in sepsis, as it was markedly lower in nonsurvivors than in survivors, and it did not change significantly over time in either of the groups. We did not find a difference in the urine MIF levels in association with the presence or absence of renal dysfunction.

Sepsis continues to constitute a serious burden for patients and a significant challenge for the healthcare system even nowadays due to its high incidence, potentially fatal outcome, and substantial costs of care^2,4^. One way to mitigate the burdens of sepsis is to discover biomarkers, which can be used for the diagnosis and for the prediction of the outcome of the disease. In accordance with that approach, a plethora of sepsis biomarker candidates were proposed (for reviews, see^38,39^), which also included MIF as a promising biomarker^6^. MIF is a multifaceted cytokine playing diverse roles in the host immune response to infectious and non-infectious stimuli^40^. It underlines the importance of MIF biology in sepsis that variant MIF alleles have been linked to altered MIF expression and Gram-negative bacteremia^41^, and that MIF levels in sepsis have previously been shown to correlate with APACHE II scores^42^. Our recent meta-analysis suggested that serum MIF level can serve as a valuable diagnostic and predictive biomarker in sepsis^7^. However, previous studies about its kinetics were scarce and reported controversial results^8–10^, even though the importance of some other biomarkers’ kinetics in sepsis has been recognized and investigated in recent studies^14,43,44^. To shed more light on the temporal changes of serum MIF in sepsis, in the present study we report its absolute levels on the initial days after ICU admission in septic patients, who were also divided into subgroups based on age, sex, and survival.

The serum MIF kinetics clearly differed between sepsis survivors and nonsurvivors after ICU admission, since in the nonsurvivors serum MIF increased, whereas in survivors it decreased. Considering that we did not always detect statistically significant difference between the outcome groups when only single measurements were compared, the novel finding about the distinct kinetics indicates that repeated serum MIF level measurements in the same patient can be better predictors of the outcome than single time-point measurement at the ICU. Indeed, the significant prognostic value of MIF was not found in some previous studies, in which the authors performed only one measurement of its serum level^45–47^. The increasing levels of serum MIF associated with the fatal outcome can be assumed to be related to the progression of the disease. MIF is a proinflammatory cytokine that promotes the immune response to defeat the pathogen^5^, which can explain why higher levels were found in patients with systemic inflammation in several studies (for review, see^7^). However, when the pathogen load is excessive or the anti-inflammatory response is depleted, the proinflammatory response can be overtly activated and become harmful for the host. The gradually increasing serum MIF level may serve as a marker for the excessively intensifying proinflammatory activity, which can be an early warning sign for healthcare personnel to initiate more aggressive treatments before fatal consequences develop. It should be noted also that MIF is present in different cell types in pre-formed, intracytoplasmic pools^47–52^, thus its increasing levels may also reflect escalating tissue damage and necrosis.

Interestingly, in survivor and deceased females the patterns of serum MIF kinetics were somewhat different from males. In females, the level of MIF increased in both groups, though the extent of the increase tended to be markedly greater in nonsurvivors than in survivors (*p* = 0.13). Similar to males, the bigger increase also developed in the deceased patients, which was, however, more pronounced in deceased females than males (651 ± 258 vs. 313 ± 207 pg/ml) (for details, see Fig. 3B). Furthermore, in the survivors there was an increase in females instead of the decrease observed in males. The intersex difference in the serum levels of MIF in the septic patients can be due to the influence of sex hormones. In experimental models of inflammation, estrogen reduced the production of MIF^34–36,53^. In accordance, MIF levels in the plasma were lower in female than in male healthy human subjects^37,54^. It should be noted, however, that estrogens were inactive when MIF was abundantly present in one of the models^53^, and that the difference in MIF levels between males and females was only present in the younger than 55 years old age group in the study by Aliosi et al.^37^. As part of the inflammatory response, MIF is rapidly produced and released into the bloodstream in sepsis^5^, thus its concentrations can be high enough to overcome the suppressive effect of estrogen on its production. With regards to age, in our study the average age of the patients was 66 ± 2 years and the youngest woman was 47 years old. It can be assumed that the majority of the included females were already in the postmenopausal period, therefore had low estrogen levels. Indeed, in a previous study the plasma concentration of estradiol in males were significantly higher than in postmenopausal women^55^. Taken together, the abundance of MIF in the bloodstream in sepsis and the decreased estrogen levels in postmenopause can serve as a hypothetical reason why the MIF levels increased in both survivor and nonsurvivor septic females to a greater extent than in males in our study. The intersex differences in serum MIF levels in sepsis can be a contributing factor to the previously reported different prognosis between septic males and females^33^. It should be mentioned also that the prognostic discrepancy between MIF levels in males and females may have a genetics basis, which is supported by differences in the statistical association between variant MIF alleles and sex in the inflammatory disease multiple sclerosis^56^.

Besides serum MIF, urine MIF level was also proposed as a disease biomarker^6^. Accordingly, its role was studied in kidney injury due to a variety causes^15–18,57^, but, to our surprise, we could not find data in the literature about the kinetics of urine MIF in sepsis and its association with sepsis-related kidney injury. In the present study, we show that urine MIF remains relatively constant on the initial days after ICU admission in both survivors and nonsurvivors. Importantly, however, in the deceased patients it was markedly lower than in survivors. These findings suggest that urine MIF can be an easily accessible biomarker for prediction of the outcome in sepsis. Due to its relatively stable levels over time, a random measurement on any days could be possibly used in practice. This is also supported by the finding that there was a strong correlation between the first and last measured levels of urine MIF in the present study. An obvious question related to urine MIF is how its levels are influenced by acute kidney injury, which is a common complication in critically ill patients at the ICU^58^. When we compared urine MIF levels between patients with and without renal dysfunction, urine MIF levels were similar in the two groups on all days and there was no difference in the kinetics and overall change in its level over time. This is in harmony with the results of an earlier study showing that the progression of renal injury was independent from renal MIF expression in a mouse model of nephropathy^59^. Our results suggest that urine MIF can be used as a predictive biomarker in sepsis independently from the kidney function, however, it does not indicate the development of sepsis-associated acute kidney injury.

The lower urine MIF level in the nonsurvivors was an unexpected new finding, which requires discussion. The increasing serum levels of MIF seem to contradict the lower urine MIF levels in patients who died in sepsis, but it can be explained by the diverse source and complex role of MIF in inflammation. Besides immune cells, MIF is produced in most cells in the kidney, e.g., tubular cells, podocytes, mesangial and endothelial cells (for recent review, see^60^). MIF is constitutively expressed in kidney tissues at low levels, but it is markedly upregulated in disease conditions such as kidney inflammation^61^. Urine MIF level showed an inferior correlation with serum MIF in a previous study^62^, indicating that its concentration in the urine is not only influenced by clearance of serum MIF, but also by its renal synthesis. Furthermore, it should be mentioned that differences in protein permeability through the glomerular basement membrane and in protein reabsorption by tubular epithelial cells may be also associated with urinary excretion of MIF in nephropathy^63^. The lack of correlation between serum and urine levels of MIF can also explain why higher serum levels were not accompanied by increased urine levels in nonsurvivors in the present study. Renal MIF was shown to possess a renoprotective function in different kidney diseases, also including acute kidney injury^64–66^. Since the urine MIF level in sepsis survivors was higher than that of deceased patients in the present study, it can be speculated that the endogenous renoprotective effect of renal MIF was attenuated in the nonsurvivor group, thereby indicating the increased severity of the disease. While the described scenario might be a possible explanation for our current findings, it should be mentioned that a causative role for MIF in the development of kidney injury was also proposed by previous studies^67–69^. The disease context and the different roles of MIF in disease pathogenesis were suggested as the causes for the contradictory (i.e., renoprotective vs. detrimental) roles in the different studies^64^. Future studies are warranted to reveal the exact function of renal MIF in sepsis.

Limitations of our study must be also mentioned. Our sample size was relatively small, which resulted in low number of patients after dividing the population into multiple subgroups (e.g., survivor males and females). The patients were enrolled at a single clinical center in the present study, thus further studies at multiple (preferably international) centers are needed to improve diversity of the patients and allow for conclusions in broader population. In the present study, we focused on patients admitted to the ICU, however, it would be also important to see how MIF kinetics develop in septic patients before the ICU admission, which could help physicians to get an insight about the prognosis at an earlier stage of the disease. Lastly, we did not correlate the kinetics of MIF levels with other biomarkers, therefore the prognostic performance of MIF could not be compared with other markers. However, Kofoed et al.^70^ showed that MIF performed similarly as procalcitonin, C-reactive protein, and neutrophil count in the detection of a bacterial cause in systemic inflammation as indicated by the areas under the receiver operating characteristics curve (AUROC) of 0.63, 0.72, 0.81, and 0.74, respectively^70^. In the same study, the measurement of combination of all these four with two other biomarkers (suPAR and sTREM-1) was found to be more useful (with AUROC of 0.88) than that of the single markers. In another report, plasma levels of MIF, procalcitonin, interleukin (IL)-6, -8, -10, and thioredoxin were elevated in patients with systemic inflammation, however, in neutropenic sepsis, MIF and thioredoxin levels were lower, whereas IL-8 and procalcitonin levels were higher compared to sepsis without neutropenia^71^. Since no correlation was found between MIF and leukocyte cell counts in that study^71^, the authors concluded that the severely reduced leukocyte number was unlikely to cause decreased MIF levels in the neutropenic patients. In contrast, there was a trend toward a positive correlation between MIF levels and leukocyte counts in another study, which finding was in agreement with the authors’ observation of low MIF levels in a neutropenic patient^10^. With regards to the prediction performance of fatal outcome in sepsis, the AUROC was found to be 0.79 for MIF and 0.68 for IL-6^72^. Significant correlations were shown between MIF and IL-6 levels and disease severity scores in septic patients, whereas no relation was found between MIF and markers of the acute phase response (procalcitonin, C- reactive protein, and lipopolysaccharide-binding protein)^73^. Finally, in certain infections, the serum level of MIF was a better biomarker than C-reactive protein or IL-6 for predicting death^74^. Taken together, the investigation of the exact correlation of serum and urine MIF level kinetics with those of other biomarkers remains subject for future studies. Nevertheless, based on the present and previous findings, the changes in MIF levels alone or in combination with other biomarkers can be useful in the diagnosis of sepsis and in prediction of the outcome. For example, it was proposed that the continuous and combined monitoring of MIF and procalcitonin levels may be useful to distinguish patients suffering from post-burn inflammation from those that will develop fatal systemic inflammation or sepsis^75^.

To the best of our knowledge, this is the first study that reports the kinetics of serum and urine MIF in septic patients admitted to the ICU. In summary, we showed that an increasing serum MIF pattern was characteristic for patients who died in sepsis, whereas the level was rather decreasing in those who survived. Intersex differences in the serum MIF level kinetics were also revealed. Last, we showed that urine MIF level was not associated with renal dysfunction and it was lower in nonsurvivors than in survivors of sepsis. Despite of its limitations, our study highlights the biomarker value of serum and urine MIF kinetics for the prediction of the outcome of sepsis. Our results can also serve as an encouraging basis for designing future studies at multinational level, which are required to determine the real prognostic value and clinical feasibility of repeated MIF level measurements in septic patients. The aims of such desirous studies could be also extended to investigate the role of the MIF congener MIF-2, which signals through the same cognate receptor (CD74), and measures of sCD74, both of which have been measured in clinical studies of other conditions^42,76,77^.

## Data Availability

All data generated or analyzed during this study are included in this published article.
